# HUE: The hourly usage of energy dataset for buildings in British Columbia

**DOI:** 10.1016/j.dib.2019.103744

**Published:** 2019-03-15

**Authors:** Stephen Makonin

**Affiliations:** Engineering Science, Simon Fraser University, Canada

## Abstract

Having access to long-term consumption data from multiple houses help research simulate and test systems for microgrid, off-grid communities, and alternative energy production. The HUE dataset contains donated data from residential customers of BCHydro, a provincial power utility. There are currently twenty-two houses contain within the dataset with most houses having three years of consumption history. Data was downloaded from BCHydro's customer web porthole by each customer who then donated the data for research. Weather data from the nearest weather station and one-year of simulated solar data also included.

**Table:** Specifications table

Subject area	*Engineering, Computer Science*
More specific subject area	*14.1: Energy::Energy (General)*
Type of data	*comma-separated-value (CSV) time-series*
How data was acquired	*Microscope, survey, SEM, NMR, mass spectrometry, etc.; if an instrument was used, please give the model and make.*
Data format	*raw*
Experimental factors	*data was anonymized by removing sensitive columns*
Experimental features	*data was donated by customer of a provincial power utility*
Data source location	*British Columbia, Canada*
Data accessibility	https://doi.org/10.7910/DVN/N3HGRN
Related research article

**Table:** 

**Value of the data** • Provides long-term consumption data from multiple houses help research simulate and test systems for microgrid and off-grid communities. • Provides real-world consumption data for developing and testing optimal energy production for communities using alternative energy or control their own energy production. • Provides data that can be used to provide financial analysis for installing battery storage for solar producing communities. • Provides data that can be used to provide financial analysis and impact analysis for simulating communities using electric vehicles.

## 1 Data

Consumption history data, simulated solar data, and weather data is stored in simple comma-separated-value (CSV) files. The summary data is stored in a fixed-length format to may it easy to read. There are a total of 27 files in this dataset. Table 1 describes the files within the dataset. Data frequency for all files is hourly (in local Pacific timezone). Data was downloaded from BCHydro's customer web porthole by each customer who then donated the data for research. Weather data was downloaded from the nearest Environment Canada [5] weather station. Simulated solar data was generated by a tool provided by the US Department of Energy [4].

**Table 1:** Filename descriptions.

Filename	Description
All_Residential.txt	Summary data for each house in listed in a table by house ID.
Holidays.csv	Indicated what days are statutory holidays etc.
Residential_<#>.csv	Hourly consumption history for each house where <#> is the ID of each house.
Solar.csv	One years worth of hourly simulated solar production data generated from the PVWatts online tool [4]. DC System Size was set to 4 kW with an Invert Efficiency of 96%.
Weather_<ID>.csv	Hourly weather station data where <ID> is the three-letter weather station ID listed in the summary data table.

## 2 Experimental design, materials, and methods

Data was obtained through donation by BCHydro customers. Each customer logged into BCHydro's customer web porthole and requested an export of historical hourly consumption data. The porthole only allows customers to download a maximum of three years worth of data. Only BCHydro customers were asked to donate to keep the data quality consistent. Customers were solicited through be emailing a pamphlet out to a network of family, friends, and work colleagues. The pamphlet is attached as supplementary material to the paper.

Table 2 describes the data columns found in each file of the dataset. Each house has some additional characteristic data that was collected about it (see the All_Residential.txt file) and each characteristic is described there, as well in Table 3. Note that there is no characteristic data for House 7.

**Table 2:** Data column descriptions.

Column	Description
ac_output	Solar AC energy produced in kilo-Watt-hours (kWh) after DC conversion.
date	Date of the recording in YYYY-MM-DD.
day	Day of the week; e.g., Monday.
dc_output	Solar DC energy produced in kilo-Watt-hours (kWh).
dst	Day light savings time indicator (−1 or +1 for hour adjustment).
energy_kWh	Energy consumed in kilo-Watt-hours (kWh).
holiday	Textual name of the holiday (indicates a working day off).
hour	Hour of the recording from 01 to 24.
humidity	Outside humidity in percentage (%).
pressure	Atmospheric pressure in kilopascals (kPa).
temperature	Outside ambient temperature in degrees Celsius (°C).
weather	A textual description the type of weather; e.g, Mostly Cloudy.
weekend	Boolean value to indicate weekend.

**Table 3:** House characteristics descriptions.

Column	Description
House	The house ID number.
FirstReading	The first reading date in the house's data file.
LastReading	The last reading date in the house's data file. At the end of each year, some house files will be updated with new data.
Cover	The data coverage. The percent of non-missing readings. A value of 1.000 is 100%.
HouseType	*character*: multi-level houses build before 1940 bungalow: single-level (w/basement) houses built in the 1940s and 1950s *special*: two-level houses built between 1965 and 1989 *modern*: two-/three-level houses build in the 1990s and afterwards *duplex*: two houses that share a common wall, can be side-by-side or front-back *triplex*: three houses that share common walls: top unit, front unit, and back unit *townhouse*: row houses that share one or two common walls *apartment*: hight-rise or low-rise living units *laneway*: small homes built in the backyard of the main house which open onto the back lane
Facing	What direction the house is facing. This often has an impact on house cooling durning the summer. East and West facing houses get hotter faster.
Region	The 3-letter code of the house's regional weather station. *YVR* - Vancouver and Lower Mainland area *WYJ* - Victoria and surrounding area
RUs	Rentals Units. The number of rental suites in the house. More rental suites means higher consumption.
EVs	Electric Vehicles. If there is an EV, what is the size of the battery (in kWh).
SN	Special Notes associated to that house which are listed within the file.
HVAC	A description of the HVAC systems which also has an impact on power consumption. One or a combination of: *FAGF* - forced air gas furnace *HP* - heat pump (incl. a/c) *FPG* - gas fireplace *FPE* - electric fireplace *IFRHG* - in-floor radiant heating (gas boiler) *NAC* - no a/c *FAC* - fixed a/c unit *PAC* - portable a/c unit *BHE* - baseboard heater (electric) *IFRHE* - in-floor radiant heating (electric) *WRHIR* - water radiant heat (cast iron radiators)

Residential House 1 is the same house used in the AMPds dataset [1], [2] which has 2-years of per-minute data including appliance-level consumption data; and is House 1 in the RAE dataset [3] with approximately 60-days of 1Hz including appliance-level consumption data. Residential House 2 is House 2 in the RAE dataset [3] with approximately one-year of 1Hz including appliance-level consumption data.
