# Supplementary material for: HUE: The hourly usage of energy dataset for buildings in British Columbia
Source: Data Brief. 2019 Mar 15;23:103744. doi: 10.1016/j.dib.2019.103744 (PMC6660473; doi:10.1016/j.dib.2019.103744)
Supplement: Supplementary file 1 — Multimedia component 1 [file mmc1.pdf]

Conflicts of Interest Statement

Manuscript title: HUE: The Hourly Usage of Energy Dataset for Buildings in British Columbia

The authors whose names are listed immediately below certify that they have NO affiliations with or involvement in any organization or entity with any financial interest (such as honoraria; educational grants; participation in speakers' bureaus; membership, employment, consultancies, stock ownership, or other equity interest; and expert testimony or patent-licensing arrangements), or non-financial interest (such as personal or professional relationships, affiliations, knowledge or beliefs) in the subject matter or materials discussed in this manuscript.

Author names:

Stephen Makonin

The authors whose names are listed immediately below report the following details of affiliation or involvement in an organization or entity with a financial or non-financial interest in the subject matter or materials discussed in this manuscript. Please specify the nature of the conflict on a separate sheet of paper if the space below is inadequate.

Author names:

This statement is signed by all the authors to indicate agreement that the above information is true and correct (*a photocopy of this form may be used if there are more than 10 authors*):

Author's name (typed)	Author's signature	Date
Stephen Makonin	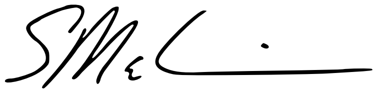	March 14, 2019
