# Supplementary material for: HUE: The hourly usage of energy dataset for buildings in British Columbia
Source: Data Brief. 2019 Mar 15;23:103744. doi: 10.1016/j.dib.2019.103744 (PMC6660473; doi:10.1016/j.dib.2019.103744)
Supplement: Multimedia component 2 [file mmc2.pdf]

How to Download 3-Years of Hourly Data From Your BCHydro Account

Purpose and Use of donating Your Data

How can you help? I need data. I am looking for homeowners to donate their historical hourly data from their BCHydro account. This only takes a matter of minutes to do. The data will be anonymized — meaning there will be no way to know it is from you. Additionally, the goal is to have the anonymized data available in a dataset for researchers around the world to test their research.

Here are just two of the project that can us your data. One project is trying to find anomalies in power usage to help predict when appliances will fail. For example, often before a fridge fails the compressor will run non-stop causing you to have a huge energy bill. The other project is looking at how installing solar panels and batteries on/in a home can save money for homeowners.

If you would like to help out, Please follow the below instruction and answer the questions int he last section. You can email the data and the answers to the questions to smakonin@sfu.ca.

Thanks!

Stephen Makonin, PhD, smIEEE
Adjunct Professor
Engineering Science, Simon Fraser University
Email: smakonin@sfu.ca
Website: http://www.sfu.ca/~smakonin/

© 2018 Stephen Makonin

Downloading Your Data

Step 1. You have to go into the accounts home/overview page:

https://app.bchydro.com/BCHCustomerPortal/web/accountsOverview.html

Step 2. Then select show all in the **MANAGE ACOUNT** box which will show a dropdown menu and select **Data export centre**

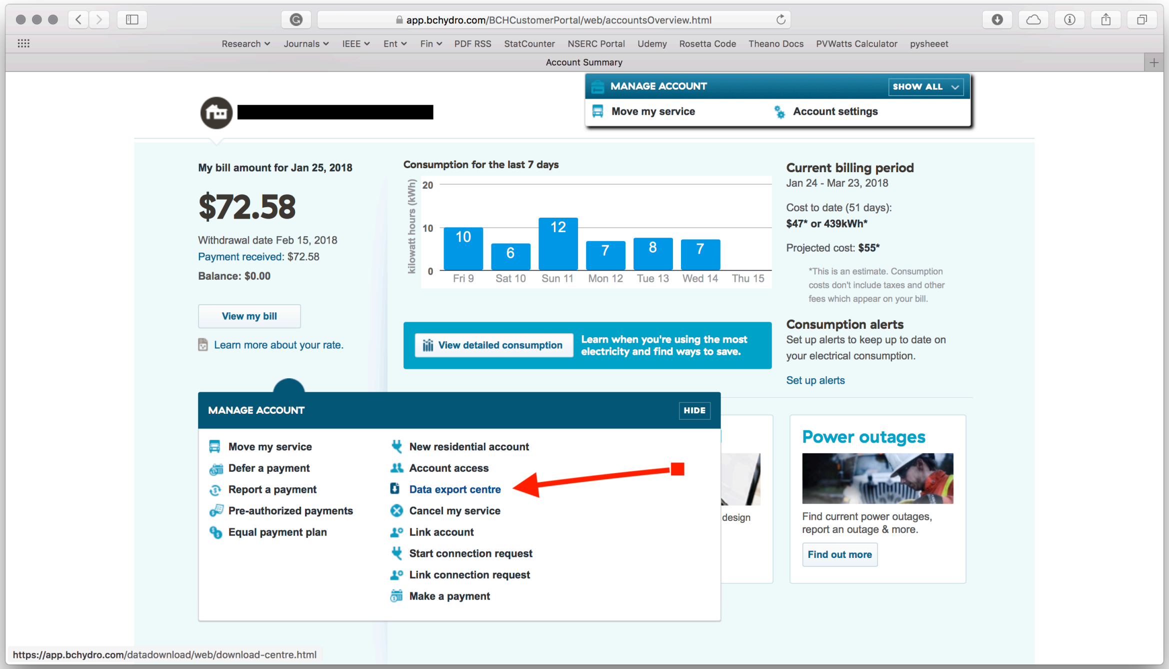

Instructions continue on next page…

© 2018 Stephen Makonin

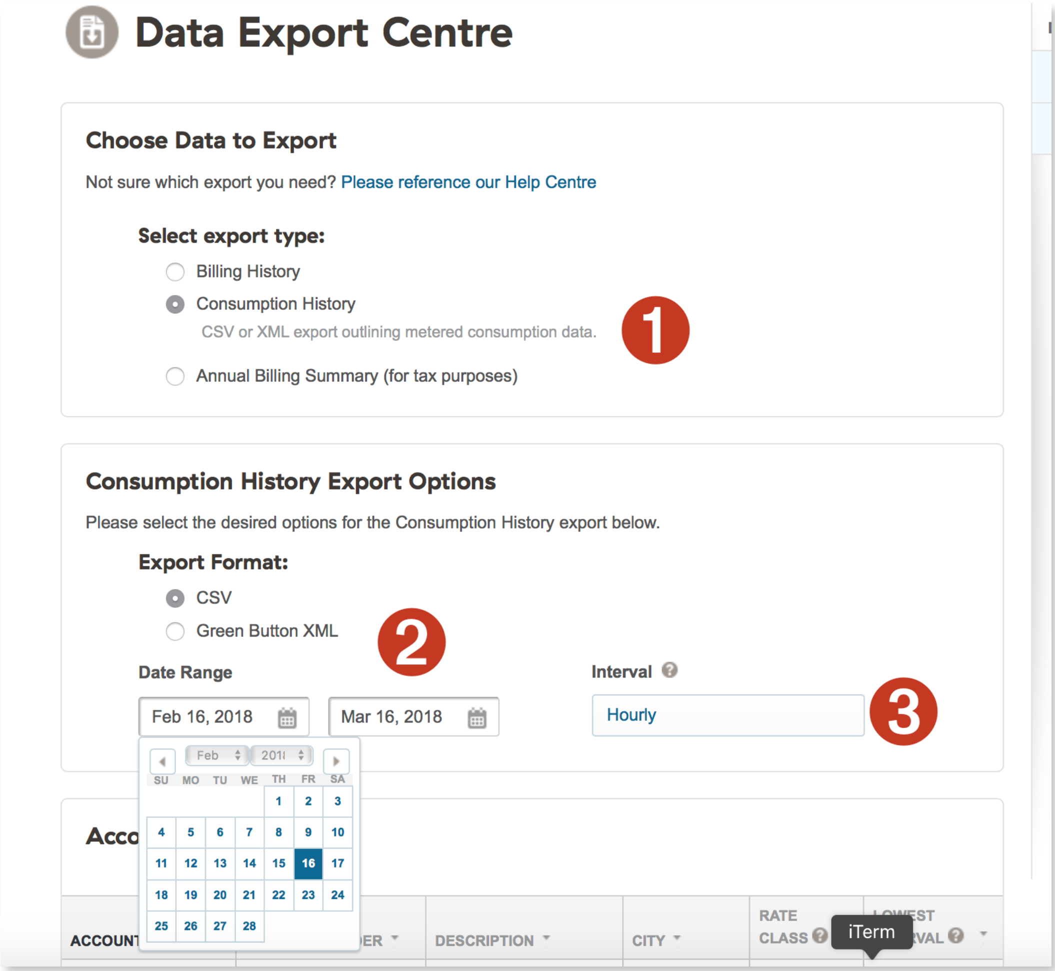

Step 3. In the next page select **Consumption History**. Leave the Export format as **CSV**.

Step 4. Select the **1st date range** should to be 3 years ago (Mar/2/2015) and the **2nd date range** should be today's date. You will need to select the from day using the dropdown calendar.

Step 5. Select the interval from **Monthly** to **Hourly**.

Step 6. Scroll down and click the **Export Data** button. There will be an alter saying:

> *Your request will be scheduled for processing within the next 24 hours. You may be able to get the data now by selecting fewer accounts or a shorter date range. Do you wish to modify your request or continue?*

Step 7. Click **continue** and you will get an email when it is finished.

© 2018 Stephen Makonin

Demographic/classification Questions

1. What is the building type?

character	- multi-level houses build before 1940
bungalow	- single-level (w/ basement) houses built in the 1940s and 1950s
special	- two-level houses built between 1965 to 1989
modern	- two-/three-level houses build in the 1990s and afterwards
duplex	- two houses that share a common wall, can be side-by-side or front-back
triplex	- three houses that share common walls: top unit, front unit, and back unit
townhouse	- row houses that share one or two common walls
apartment	- hight-rise or low-rise living units
laneway	- small homes in the backyard of the main which open onto the back lane

2. What direction is the house facing?

3. Does the house have rental suites? If so, how many?

4. Does the house have an EV car? If so, what is the battery size (in kWh)?

5. What type of heating system does the house have?

FAGF	- forced air gas furnace
HP	- heat pump (incl. a/c)
FPG	- gas fireplace
FPE	- electric fireplace
IFRHG	- in-floor radiant heating (gas boiler)
BHE	- baseboard heater (electric)
IFRHE	- in-floor radiant heating (electric)

*** If your type of heating system is not in this list please describe.

6. Does the house have A/C (air conditioning)?

HP	- heat pump (incl. a/c)
NAC	- no a/c
FAC	- fixed a/c unit
PAC	- portable a/c unit

© 2018 Stephen Makonin
